# Evaluating the predictive performance of the elderly patient calculator TIPS score in a North American cohort

**DOI:** 10.1097/HC9.0000000000000346

**Published:** 2024-01-22

**Authors:** Roy X. Wang, Francesco Vizzutti, Ciro Celsa, Filippo Schepis, David E. Kaplan, Nadim Mahmud

**Affiliations:** 1 Department of Medicine, Hospital of the University of Pennsylvania, Philadelphia, Pennsylvania, USA; 2 Department of Experimental and Clinical Medicine, University of Florence, Florence, Italy; 3 Department of Health Promotion, Mother and Child Care, Internal Medicine and Medical Specialties, PROMISE, Gastroenterology and Hepatology Unit, University of Palermo, Palermo, Italy; 4 Department of Medicine, Division of Gastroenterology, Modena Hospital, University of Modena and Reggio Emilia, Modena, Italy; 5 Department of Medicine, Division of Gastroenterology and Hepatology, Hospital of the University of Pennsylvania, Philadelphia, Pennsylvania, USA

## Abstract

**Figure:**
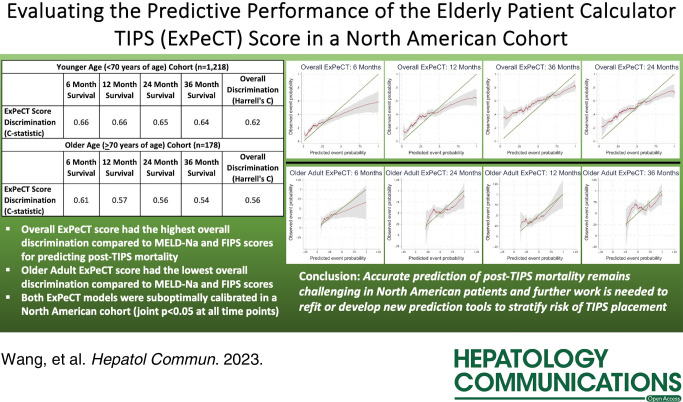

## INTRODUCTION

TIPS is an effective intervention for the treatment of portal hypertensive complications of cirrhosis, including refractory ascites and variceal bleeding. However, TIPS placement can precipitate HE and liver failure, requiring liver transplantation. Risk stratification before candidate selection is therefore critical to mitigate adverse outcomes. Models like the Model for End-Stage Liver Disease Sodium (MELD-Na) and the Freiburg index of post-TIPS survival (FIPS) have been proposed to estimate the risk of mortality post-TIPS.^1,2^ Vizzutti et al recently proposed 2 post-TIPS prediction tools for overall patients [(overall elderly patients calculator TIPS (ExPeCT)] and for patients ≥ 70 years of age (older adult ExPeCT), using an Italian cohort.^3^ Due to health differences between Americans and Europeans, it is unclear how well these models translate to North American patients.^4^ We aimed to evaluate the performance of these post-TIPS mortality prediction tools in a large North American cohort.

## METHODS

We performed a retrospective cohort study using data from the Veterans Health Administration (VHA). Patients ≥ 18 years of age from 2008 to 2022 who underwent TIPS, identified by current procedure terminology code, were included. Prior liver transplant recipients were excluded. Demographic data, including age, sex, race, body mass index, medical comorbidities, etiology of liver disease, and prior cirrhosis decompensations, were obtained using validated algorithms and methods.^5–8^ Serum sodium, creatinine, albumin, total bilirubin, international normalized ratio, and platelet count within 30 days before TIPS were collected. Post-TIPS mortality was determined at 6, 12, 24, and 36 months.^9^ MELD-Na score, FIPS score, and both ExPeCT scores were calculated as described.^1–3,7^

Descriptive statistics for the VHA cohort were presented as means with SDs for continuous variables and percentages for categorical variables. Demographic data from the FIPS and ExPeCT derivation studies were included where available for comparison. The overall cohort was stratified into patients < 70 years of age (younger cohort) and ≥ 70 years of age (older cohort) to evaluate the overall and older adult ExPeCT models. The predictive ability of the MELD-Na, FIPS, and both ExPeCT scores was assessed through discrimination and calibration.^10^ Discrimination through maximum follow-up time was evaluated by Harrell’s C. Discrimination at 6, 12, 24, and 36 months post-TIPS was assessed using a time-dependent AUC computed at the specified timepoints from univariable Cox regression models. Calibration of risk scores was evaluated by plotting observed events versus predicted probabilities and calculating the intercept (β_0_) and slope (β_1_) of the fitted regression line. Joint hypothesis tests evaluating a null hypothesis of β_0_=0 and β_1_=1 were performed at the specified timepoints with *p*-value<0.05, signifying poor calibration. Institutional Review Board approval was obtained from the Michael J. Crescenz Philadelphia Veterans Affairs Medical Center. All dataset manipulation and statistical analyses were performed using STATA 17.0/SE (College Station, TX).

## RESULTS

The final cohort included 1218 patients in the younger cohort and 178 patients in the older cohort (Supplemental Table S1, http://links.lww.com/HC9/A763). Supplemental Figure S1, http://links.lww.com/HC9/A763 depicts trends in TIPS placement and shows TIPS placement in the older cohort was more common after 2016. Both cohorts were predominantly male and White. The older cohort had a higher proportion of metabolic dysfunction–associated steatotic liver disease as the etiology of cirrhosis (33.1% vs. 12.9%, *p* < 0.001). Metabolic comorbidities were more prevalent in the older cohort. Rates of prior decompensations were similar between the older (94.4%) and younger cohorts (94.2%) (*p* = 0.91). MELD-Na score at TIPS was significantly higher in the younger cohort (14.6 vs. 13.2, *p* = 0.01). The older cohort had higher post-TIPS mortality at 12, 24, and 36 months (each *p* < 0.05).

The predictive performance of the FIPS, MELD-Na, and ExPeCT scores are shown in Table 1. The overall ExPeCT score had the highest overall discrimination in the younger cohort, with Harrell’s *C* of 0.62. In the older cohort, the older adult ExPeCT score had the lowest overall discrimination of 0.56. Calibration curves of prediction scores for each cohort are presented in Supplemental Figures S2, http://links.lww.com/HC9/A763 (younger cohort) and S3, http://links.lww.com/HC9/A763 (older cohort). There was evidence of poor calibration for the ExPeCT scores at all timepoints (joint *p* < 0.05). In evaluating calibration curves, the ExPeCT scores tended to overestimate post-TIPS mortality in higher-risk patients and underestimate risk in lower-risk patients.

**TABLE 1:** Discrimination and calibration of prediction scores

	6 mo survival	12 mo survival	24 mo survival	36 mo survival	Overall discrimination (Harrell’s C)
Younger age cohort (n = 1218)					
FIPS					
Discrimination (AUC)	0.66	0.63	0.62	0.61	0.60
Intercept (95% CI)	−0.01 (−0.15, 0.13)	−0.04 (−0.16, 0.07)	−0.04 (−0.14, 0.07)	0.02 (−0.08, 0.12)	—
Slope (95% CI)	0.80 (0.62, 0.98)	0.57 (0.43, 0.71)	0.43 (0.31, 0.56)	0.35 (0.23, 0.46)	—
Joint test (*p*-value)	*p*=0.07	*p*<0.001	*p*<0.001	*p*<0.001	—
MELD-Na					
Discrimination (AUC)	0.58	0.58	0.57	0.57	0.58
Intercept (95% CI)	0.10 (−0.03, 0.22)	0.05 (−0.05, 0.15)	−0.004 (−0.09, 0.09)	−0.01 (−0.10, 0.07)	—
Slope (95% CI)	1.40 (0.67, 2.13)	1.41 (0.83, 1.99)	1.31 (0.81, 1.80)	1.30 (0.82, 1.78)	—
Joint test (*p*-value)	*p*=0.19	*p*=0.24	*p*=0.48	*p*=0.45	—
ExPeCT score					
Discrimination (AUC)	0.66	0.66	0.65	0.64	0.62
Intercept (95% CI)	0.15 (−0.01, 0.32)	0.25 (0.13, 0.38)	0.29 (0.18, 0.40)	0.38 (0.28, 0.49)	—
Slope (95% CI)	0.45 (0.33, 0.57)	0.47 (0.35, 0.59)	0.43 (0.32, 0.54)	0.38 (0.28, 0.49)	—
Joint test (*p*-value)	*p*<0.001	*p*<0.001	*p*<0.001	*p*<0.001	—
Older age cohort (n = 178)					
FIPS					
Discrimination (AUC)	0.62	0.59	0.57	0.56	0.58
Intercept (95% CI)	−0.11 (−0.41, 0.19)	−0.12 (−0.38, 0.14)	−0.05 (−0.29, 0.19)	0.08 (−0.17, 0.33)	—
Slope (95% CI)	0.92 (0.39, 1.45)	0.53 (0.10, 0.96)	0.33 (−0.04, 0.70)	0.24 (−0.12, 0.59)	—
Joint test (*p*-value)	*p* = 0.76	*p* = 0.08	*p* < 0.01	*p* < 0.001	—
MELD-Na					
Discrimination (AUC)	0.56	0.59	0.58	0.56	0.59
Intercept (95% CI)	−0.05 (−0.34, 0.24)	−0.03 (−0.27, 0.20)	−0.03 (−0.26, 0.19)	0.03 (−0.20, 0.25)	—
Slope (95% CI)	1.03 (0.18, 1.89)	1.32 (0.63, 2.01)	0.91 (0.28, 1.54)	0.66 (0.01, 1.31)	—
Joint test (*p*-value)	*p*=0.93	*p*=0.64	*p*=0.91	*p*=0.59	—
ExPeCT score					
Discrimination (AUC)	0.61	0.57	0.56	0.54	0.56
Intercept (95% CI)	0.07 (−0.27, 0.41)	0.02 (−0.25, 0.29)	0.08 (−0.17, 0.33)	0.19 (−0.06, 0.44)	—
Slope (95% CI)	0.49 (0.13, 0.84)	0.47 (0.12, 0.82)	0.37 (0.03, 0.71)	0.27 (−0.07, 0.61)	—
Joint test (*p*-value)	*p* = 0.01	*p* = 0.01	*p* = 0.001	*p* < 0.001	—

Abbreviations: ExPeCT, elderly patients calculator TIPS; FIPS, Freiburg index of post-TIPS survival; MELD-Na, Model for End-Stage Liver Disease Sodium.

## DISCUSSION

In this large retrospective study of VHA patients with cirrhosis undergoing TIPS, we found that the overall ExPeCT score had the highest discrimination of all scores in patients aged < 70 years. AUCs in our cohort at 12, 24, and 36 months (0.66, 0.65, and 0.64, respectively) were comparable to reported AUCs from the validation cohort in the ExPeCT study (0.63, 0.63, and 0.63).^3^ However, in patients aged ≥ 70 years, the ExPeCT TIPS score had the lowest overall discrimination of all scores, with AUC results somewhat worse than those reported by Vizzutti et al at 12, 24, and 36 months (0.57, 0.56, and 0.54 vs. 0.58, 0.58, and 0.58, respectively).^3^ Estimated risks of post-TIPS mortality were overly extreme for both overall and older adult ExPeCT scores, as also noted for the overall ExPeCT score in the derivation study.^3^ The observed differences in discrimination and calibration may stem from underlying differences in the North American VHA cohort as compared to the Italian cohorts, including in terms of male predominance, medical comorbidities, hepatitis C-related cirrhosis, and differences in other uncaptured patient characteristics. This suggests that tailored scores may be necessary for North American cohorts or that existing models need to be refit/recalibrated for this population.

Several key limitations exist for our study. Our cohort was predominantly male, limiting the interpretation of results for females. Given the retrospective nature of the study, there may be misclassification of exposures and outcomes. Validated VHA algorithms were used whenever possible to minimize this bias.^5–9^ Detailed data related to TIPS placement and function, such as TIPS diameters and pre/post pressure gradients, which may impact post-TIPS mortality, were unavailable in our dataset.

In conclusion, while the overall ExPeCT model had the highest discrimination of all scores in patients < 70 years of age, both ExPeCT scores had evidence of poor calibration. Accurate prediction of post-TIPS mortality remains challenging in North American patients, particularly in patients aged ≥ 70 years. Current prediction models should be used cautiously in this population, and refitting or development of novel prediction scores is needed to better risk-stratify patients for TIPS placement.

## Supplementary Material

SUPPLEMENTARY MATERIAL
