# Supplementary material for: Evaluating the predictive performance of the elderly patient calculator TIPS score in a North American cohort
Source: Hepatol Commun. 2024 Jan 22;8(2):e0346. doi: 10.1097/HC9.0000000000000346 (PMC10805419; doi:10.1097/HC9.0000000000000346)
Supplement: SUPPLEMENTARY MATERIAL [file hc9-8-e0346-s001.docx]

**Supplemental Digital Content**

**Title:** Evaluating the Predictive Performance of the Elderly Patient Calculator TIPS (ExPeCT) Score in a North American Cohort

**Authors:** Roy X. Wang MD^1^

*1. Department of Medicine, Hospital of the University of Pennsylvania, United States*

**Supplemental Table 1: Demographic Description of Younger and Older Cohort**

| **Factor** | **VHA Cohort Age <70 (n=1218)** | **VHA Cohort Age >70 (n=178)** | **p-value^a^** | **FIPS Training Cohort (n=1496)** | **ExPeCT Deriviation Cohort Age <70 (n=312)** | **ExPeCT Derivation Cohort Age >70 (n=99)** | **p-value^b^** |
| --- | --- | --- | --- | --- | --- | --- | --- |
| **Age, mean (SD)** | 59.3 (6.9) | 73.1 (3.0) | <0.001 | 57.9 (12.1) | 59.0 (5.6) | 74.0 (3.3) |  |
| **Male Sex** | 1191 (97.8%) | 174 (97.8%) | 0.98 |  | 288 (73.1%) | 69 (69.7%) | 0.60 |
| **Race** |  |  | 0.20 |  |  |  |  |
| **Asian** | 12 (1.0%) | 1 (0.6%) |  |  |  |  |  |
| **Black** | 78 (6.4%) | 6 (3.4%) |  |  |  |  |  |
| **Hispanic** | 97 (8.0%) | 19 (10.7%) |  |  |  |  |  |
| **Other** | 128 (10.5%) | 13 (7.3%) |  |  |  |  |  |
| **White** | 903 (74.1%) | 139 (78.1%) |  |  |  |  |  |
| **BMI, mean (SD)** | 28.8 (6.3) | 28.2 (4.8) | 0.19 |  |  |  |  |
| **Etiology of Liver Disease** |  |  | <0.001 |  |  |  |  |
| **MASLD** | 157 (12.9%) | 59 (33.1%) |  | 38 (2.5%) | 56 (17.9%) | 17 (17.2%) | 0.50 |
| **Alcholic Related Liver Disease (ALD)** | 569 (46.7%) | 82 (46.1%) |  | 967 (64.6%) | 130 (41.7%) | 18 (18.2%) | <0.001 |
| **Hepatitis C Virus (HCV)** | 98 (8.0%) | 10 (5.6%) |  | 131 (8.8%) | 57 (18.3%) | 38 (38.4%) | <0.001 |
| **Hepatitis B Virus** | 11 (0.9%) | 4 (2.2%) |  | 43 (2.9%) | 13 (4.2%) | 3 (3.0%) | 0.53 |
| **ALD + HCV** | 351 (28.8%) | 16 (9.0%) |  |  | 15 (4.8%) | 3 (3.0%) | 0.63 |
| **Other** | 32 (2.6%) | 7 (3.9%) |  | 317 (21.2%) | 40 (12.8%) | 19 (19.2%) |  |
| **Diabetes** | 789 (64.8%) | 130 (73.0%) | 0.03 |  | 35 (11.2%) | 13 (13.1%) | 0.08 |
| **Coronary Artery Disease** | 259 (21.3%) | 72 (40.4%) | <0.001 |  |  |  |  |
| **Heart Failure** | 144 (11.8%) | 39 (21.9%) | <0.001 |  |  |  |  |
| **Atrial Fibrillation** | 75 (6.2%) | 30 (16.9%) | <0.001 |  |  |  |  |
| **Prior Cirrhosis Decompensation** | 1147 (94.2%) | 168 (94.4%) | 0.91 |  |  |  |  |
| **Prior Hepatic Encephalopathy** | 405 (33.3%) | 47 (26.4%) | 0.07 | 195 (13.0%) | 60 (19.2%) | 8 (8.1%) | 0.02 |
| **Prior Varices** | 853 (70.0%) | 117 (65.7%) | 0.24 |  |  |  |  |
| **Prior Ascites** | 841 (69.0%) | 119 (66.9%) | 0.56 | 1131 (75.6%) |  |  |  |
| **MELD-Na, mean (SD)** | 14.6 (6.6) | 13.2 (6.0) | 0.01 | 16.0 (6.0) | 13.9 (4.4) | 13.5 (4.6) | 0.48 |
| **CTP Score, mean (SD)** | 6.8 (1.3) | 6.4 (1.1) | <0.001 | 8.0 (2.0) | 7.6 (1.4) | 7.3 (1.3) | 0.08 |
| **CTP Class** |  |  | <0.001 |  |  |  | 0.02 |
| **Class A** | 544 (44.7%) | 109 (61.2%) |  | 250 (16.7%) | 62 (19.8%) | 26 (26.3%) |  |
| **Class B** | 628 (51.4%) | 67 (37.6%) |  | 974 (65.1%) | 222 (71.3%) | 70 (70.7%) |  |
| **Class C** | 48 (3.9%) | 2 (1.1%) |  | 272 (18.2%) | 28 (9.0%) | 3 (3.0%) |  |
| **Sodium, mean (SD)** | 136.2 (4.6) | 137.1 (4.3) | 0.01 | 135.0 (6.0) | 136 (5) | 137 (5) | 0.07 |
| **Creatinine, mean (SD)** | 1.2 (0.8) | 1.30 (0.6) | 0.12 | 1.4 (0.2) | 1.04 (0.45) | 1.19 (0.53) | 0.02 |
| **Albumin, mean (SD)** | 2.9 (0.60) | 3.1 (0.6) | 0.02 | 3.0 (0.7) | 3.32 (0.58) | 3.36 (0.55) | 0.56 |
| **Total Bilirubin, mean (SD)** | 2.1 (2.3) | 1.5 (1.1) | <0.001 | 1.8 (2.1) | 1.41 (0.81) | 1.27 (0.75) | 0.11 |
| **INR, mean (SD)** | 1.4 (0.3) | 1.3 (0.2) | <0.01 | 1.2 (0.2) | 1.32 (0.2) | 1.26 (0.18) | 0.01 |
| **Platelets, mean (SD)** | 122.0 (70.0) | 120.4 (59.7) | 0.76 | 143.0 (90.0) | 107 (83) | 98 (46) | 0.19 |
| **Mortality at 6 months** | 246 (20.2%) | 47 (26.4%) | 0.06 | 318 (21.3%) |  |  |  |
| **Mortality at 12 months** | 362 (29.7%) | 69 (38.8%) | 0.02 |  | 37 (12%)***** | 19 (19%)***** |  |
| **Mortality at 24 months** | 506 (41.5%) | 91 (51.1%) | 0.02 |  | 44 (14%)***** | 30 (30%)***** |  |
| **Mortality at 36 months** | 602 (49.4%) | 104 (58.4%) | 0.03 |  | 65 (21%)***** | 41 (41%)***** |  |

**^a^**Comparison between VHA Cohort <70 and >70 years of age

**^b^**Comparison between ExPeCT Cohort <70 and >70 years of age

*****Liver related mortality

**Supplemental Figure 1: Trends in TIPS Utilization Over Time by Age Group**

**
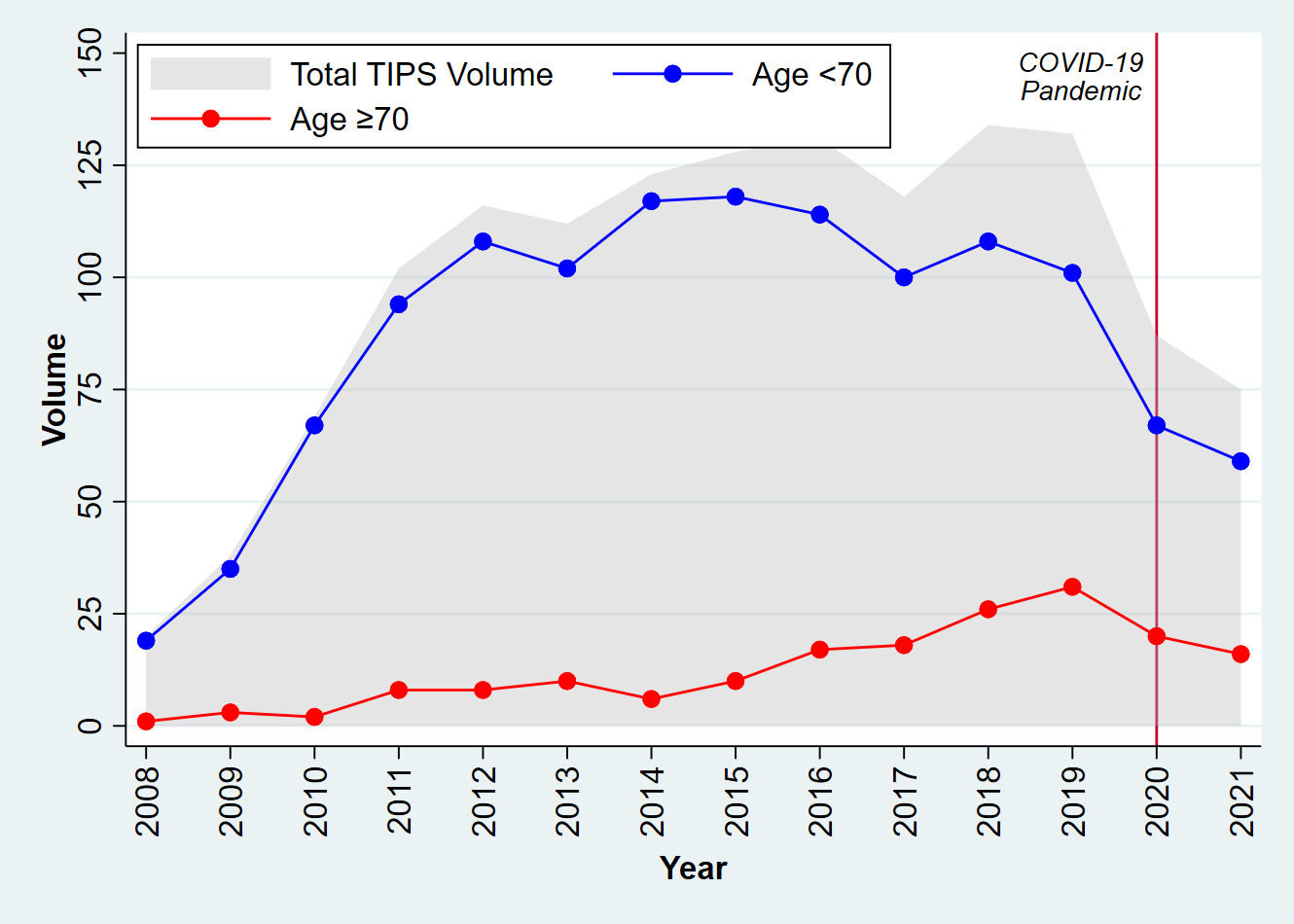
**

**Supplemental Figure 2: Calibration Curves of Prediction Scores in Younger Cohort (A) FIPS Score Calibration Curves at 6, 12, 24, 36 months post-TIPS, (B) MELD-Na Score Calibration Curves at 6, 12, 24, 36 months post-TIPS, and (C) Overall ExPeCT Score Calibration Curves at 6, 12, 24, 36 months post-TIPS**

**
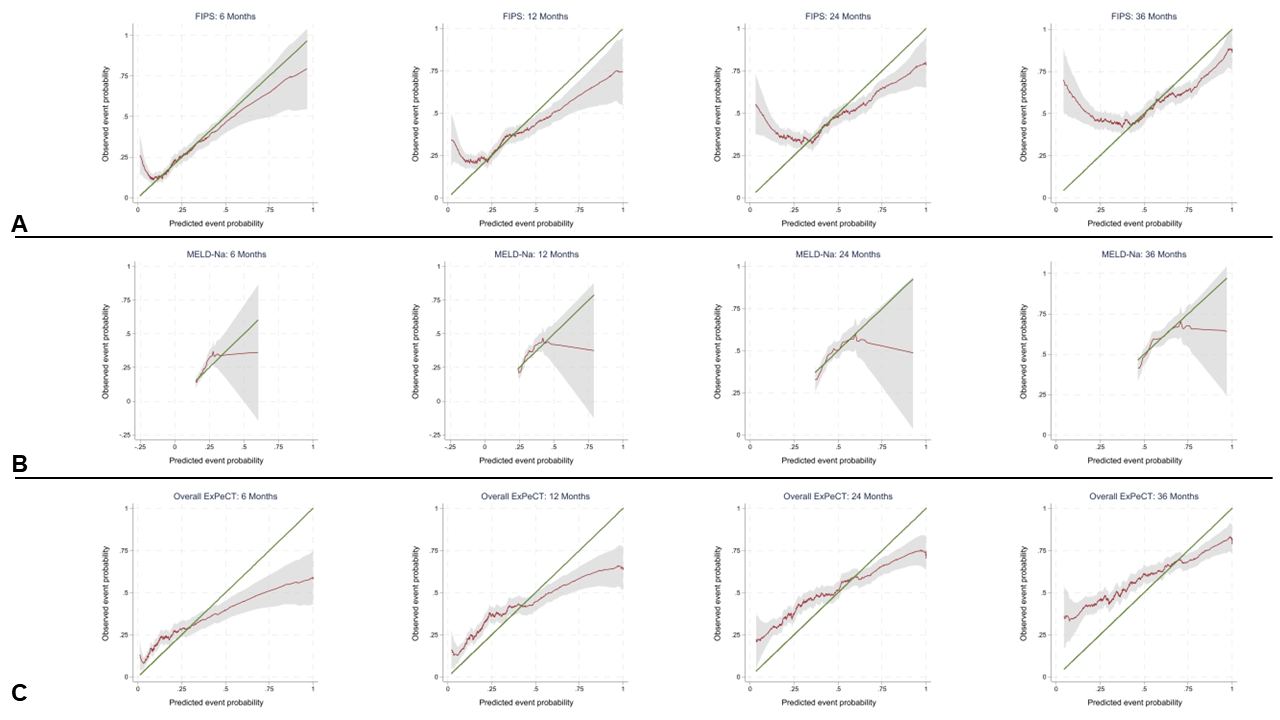
**

**Supplemental Figure 3: Calibration Curves of Prediction Scores in Older Adult Cohort (A) FIPS Score Calibration Curves at 6, 12, 24, 36 months post-TIPS, (B) MELD-Na Score Calibration Curves at 6, 12, 24, 36 months post-TIPS, and (C) Older Adult ExPeCT Score Calibration Curves at 6, 12, 24, 36 months post-TIPS**

**
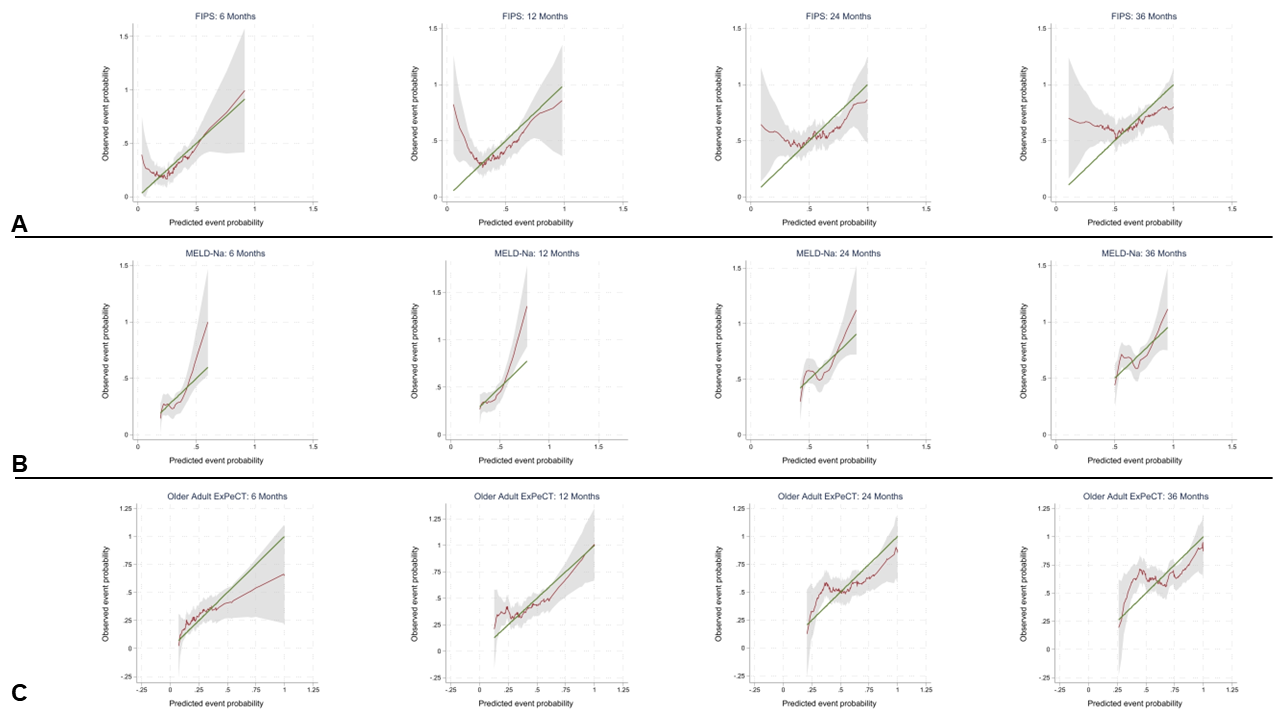
**
